# Estimating 24-h urinary sodium excretion from casual spot urine specimen among hypertensive patients in Northwest China: the Salt Substitute and Stroke Study

**DOI:** 10.1017/S1368980019005019

**Published:** 2020-04-29

**Authors:** Yi Zhao, Wanlu Liu, Sha Liu, Xiaoxia Li, Ting Yin, Xiuying Liu, Faxuan Wang, Xiaoyu Chang, Tianjing Zhang, Maoyi Tian, Yuhong Zhang

**Affiliations:** 1 Public Health and Management School of Ningxia Medical University, Yinchuan City, Ningxia, China; 2 The People’s Hospital of Anyang City, Anyang City, Henan Province, China; 3 The George Institute for Global Health at Peking University Health Science Center, Haidian District, Beijing 100088, China; 4 The George Institute for Global Health, University of New South Wales, Newtown, NSW 2042, Australia

**Keywords:** 24-h urine, Casual spot urine, Sodium, Hypertensive patients, Equation

## Abstract

**Objective:**

To develop an equation that can estimate the 24-h urinary Na excretion by using casual spot urine specimen for older hypertensive participants in rural Ningxia and further to compare with the INTERSALT method, Kawasaki method and Tanaka method.

**Design:**

Older hypertensive participants in rural Ningxia provided their casual spot urine samples and 24-h urine samples between January 2015 and February 2017. Sex-specific equation was developed using linear forward stepwise regression analysis. Model fit was assessed using adjusted *R*^2^. Approximately half of all participants were randomly selected to validate the equation. Mean differences, intraclass correlation coefficients and Bland–Altman plots were used to evaluate the performance of all methods.

**Setting:**

Pingluo County and Qingtongxia County in Ningxia Hui Autonomous Region, China.

**Participants:**

Older hypertensive participants in rural Ningxia.

**Results:**

Totally, 807 of 1120 invited participants provided qualified 24-h urine samples and spot urine samples. There was no statistical difference comparing the laboratory-based method against the new method and the INTERSALT method, while Kawasaki method had the largest bias with a mean difference of 40·81 g/d (95 % CI 39·27, 42·35 g/d). Bland–Altman plots showed similar pattern of the results.

**Conclusion:**

The INTERSALT method and the new equation have the potential to estimate the 24-h urinary Na excretion in this study population. However, the extrapolation of the results to other population needs to be careful. Future research is required to establish a more reliable method to estimate 24-h urinary Na excretion.

High Na intake is a modifiable risk factor for hypertension. Studies showed every 100 mmol/d of Na intake resulted in 5·8 and 3·8 mmHg increases in systolic and diastolic blood pressure, respectively^(1,2)^. Excessive Na intake is also associated with cerebrovascular diseases and gastric cancer^(3,4)^. World Health Organization recommended the daily Na intake within 2 g (equivalent to 5 g of salt per d) for adults^(5)^. There is an urgent need to establish a feasible solution to monitor the Na intake on a population level. 24-h dietary recall and 24-h urine collection remain as the two widely used methods to estimate population Na intake. 24-h dietary recall usually underestimated Na intake due to under-reporting and intangible Na content in food^(6)^. 24-h urine collection is considered as the gold standard to assess dietary Na intake^(7)^, but it is expensive in large-scale population survey and burdensome to participants. Previous studies used casual spot urine specimen to replace 24-h urine to assess dietary Na intake^(8–10)^. INTERSALT method, Kawasaki method and Tanaka method have been established to estimate 24-h urinary Na excretion from spot urine^(11–13)^. However, whether these three methods are valid to Chinese rural hypertensive patients remains unknown.

Therefore, the objectives of this study are to develop a method to estimate 24-h urinary Na excretion by using casual spot urine specimen in rural hypertensive patients in Ningxia Hui Autonomous Region (thereafter ‘Ningxia’) and further to compare this method with the other three established methods.

## Methods

### Study sites and participants

This study was a sub-study of the current ongoing large-scale cluster randomised controlled trial: the Salt Substitute and Stroke Study. The trial was registered in clinicaltrial.gov (NCT02092090). A total of 120 villages from Pingluo County and Qingtongxia County in Ningxia participated in the Salt Substitute and Stroke Study trial. In each village, thirty-five individuals with high risk of stroke were recruited. The selection criteria of the participants included: (1) with a history of stroke; (2) or age ≥ 60 years with uncontrolled high blood pressure (systolic blood pressure ≥ 140 mmHg at visit if on blood pressure lowering medication or systolic blood pressure ≥ 160 mmHg at visit if not on blood pressure lowering medication). A series of process indicators were measured in a random subset of individuals. Key indicators include blood pressure, medication use, urinary Na and urinary K measured from the 24-h urine and spot urine samples. The process indicators were collected from a random sample of twenty individuals drawn from a stratified random sample of at least six villages in each county (three from the intervention group and three from the control group). In each village, a randomised list of participants was generated. Interviewers invited the first twenty participants from the list to participate the survey. If anyone from the list was unable to take part in, the interviewer will move to the next available participant. Participants with the following conditions were excluded from the urine collection: (1) urinary incontinence; (2) unable to collect urine on its own and cannot find help from others; (3) acute or chronic urinary tract infection, vaginal infection and perianal infection; (4) acute or chronic urinary tract, vaginal and gastrointestinal bleeding; (5) females who were pregnant, breast-feeding or during menstruation (only allowed to participate after 2 d when their period was clear); (6) vomiting or diarrhoea. The process indicator survey was repeated every 12 months throughout the follow-up. Details of the Salt Substitute and Stroke Study trial were published elsewhere^(14,15)^.

### Specimen collection and analysis

At each process indicator survey, participants were invited to collect both casual spot urine and 24-h urine samples. Participants were asked to provide a casual spot urine specimen (midstream urine) before the collection of 24-h urine. The spot urine was collected into a disposable urine cup, and two 1·5 ml aliquots were removed. 24-h urine collection started immediately after the spot urine collection completed. The participants were provided with six 1-litre plastic containers and instructed to collect all urine voided during a 24-h period according to standard procedures. Participants were asked to return all containers at the completion of the 24-h urine collection. Interviewers recorded the start and end time of 24-h urine collection, the total urine volume and participant’s self-reported missed volume on a worksheet. 24-h urine specimens were excluded in any of the following conditions: (1) the first void of urine in the morning was missed; (2) the missed volume reported by the participant was over 10 % of the total volume; (3) the urine was contaminated or (4) the participant reported vomiting or diarrhoea during the collection. In addition, samples were excluded from the analysis if 24-h creatinine excretion was <4 mmol or >25 mmol in women or <6 mmol or >30 mmol in men.

The fieldwork was carried out between January 2015 and February 2017. Both spot urine and 24-h urine specimens were frozen at –20°C within 4 h after collection. Specimens were then shipped to a central laboratory in Beijing for analysis. The concentration of urinary Na and K was analysed using ion selective electrode method, and urinary creatinine was analysed using sarcosine oxidase method with the HITACHI 7600 automated biochemistry analyzer. Weight, height and blood pressure were measured according to a standardised protocol. Participants’ demographic information was also collected.

### Statistical analysis

Continuous variables were described as mean ± sd, while categorical variable were described as proportions (%). Spot urine Na concentration (Na_spot_), spot urine K concentration (K_spot_), spot urine creatinine concentration (Cr_spot_), age, weight, height and BMI were used in the regression analysis to develop the new method. Age, weight, height and BMI are influence factors on volume of urinary, and creatinine concentration is thought to be constant throughout the day^(16)^. The factors mentioned above were included in published predictive equations: the INTERSALT equation, Kawasaki equation and Tanaka equation^(11–13,17)^. All participants’ data were defined as the test data set to develop the sex-specific equations. Approximately 50 % of all participants were randomly selected as validation data set to verify the equations. All regression analyses were linear forward stepwise regression analysis. Adjusted *R*^2^ was used to assess the model fitting.

The performance of all methods was determined by comparing the laboratory-based method against the new method, INTERSALT method, Kawasaki method and Tanaka method. Mean difference, intraclass correlation coefficient and Bland–Altman plots were used to evaluate the agreement^(18)^. All statistical analyses were performed using SPSS (version 14.0, SPSS Corp.). Bland–Altman plots were drawn by Medcalc Software (version 15.2.2, MedCalc Software Crop.).

## Results

### Study flow chart

**Fig. 1:**
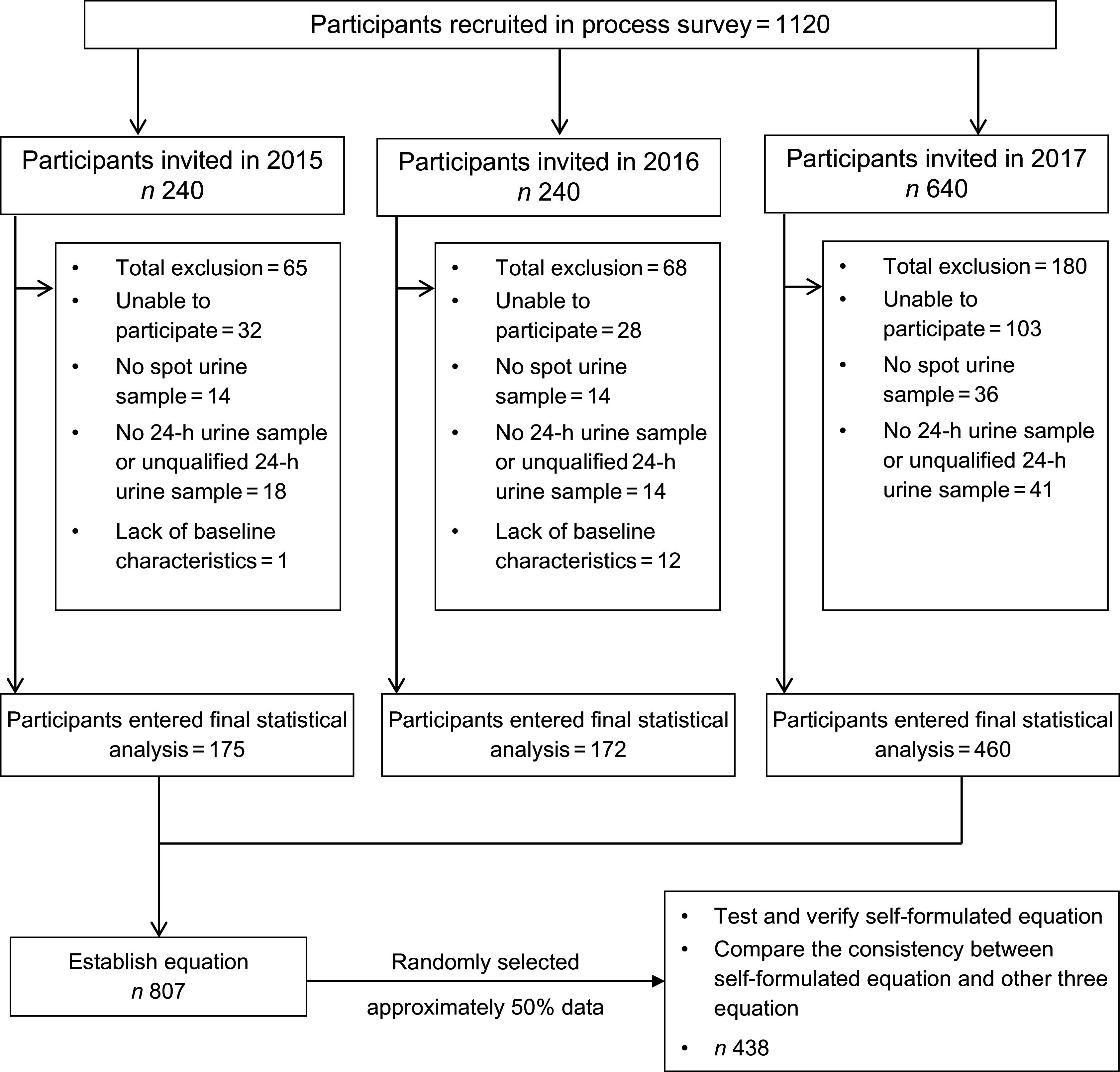
Flow chart showed the details of the study. A total of 1120 participants were invited to participate in the survey from January 2015 to February 2017. A total of 807 individuals were included in the statistical analysis because of various reasons. The PreUNa equation was established by the data from all participants. The validity of the PreUNa equation was tested by the data from 50 % randomly selected participants. PreUNa: predicted 24-h urinary Na excretion

A total of 1120 individuals recruited between January 2015 and February 2017 were invited to participate in the survey. 162 (14·46 %) refused, whereas sixty-four (6·7 %) were not able to provide casual spot urine samples and 73 (7·6 %) provided unqualified 24-h urine samples. In addition, 14 (1·5 %) individuals were excluded from data analysis due to the missing data. Finally, 807 (84·2 %) participants were included in the statistical analysis. Details are shown in Fig. 1.

### Selected characteristics of the participants

In total, a typical participant was a 68·9 years old male. Selected characteristics of the participants are shown in Supplemental Table 1.

### Regression models

Na_spot_ (mmol/l), K_spot_ (mmol/l)_,_ Cr_spot_ (mmol/l), age, height (cm), weight (kg) and BMI were used in the linear stepwise regression analysis and the gender-specific regression models. Included and removed variables were presented in Supplemental Table 2. The predicted 24-h urinary Na excretion (PreUNa) equation was as follows:

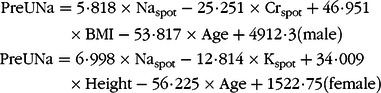

*R*^2^ was 0·137 and 0·148 for male- and female-specific equations, respectively.

### Validity of four methods

The four methods (INTERSALT^(13)^, Kawasaki^(12)^, Tanaka^(11)^ and PreUNa) are summarised in Table 1. The performance of the four methods by comparing the laboratory-based method against the four methods is presented in Table 2. In the new method, the mean estimated 24-h urinary Na excretion was higher in female compared with male (*P* = 0·09). Whereas, in the INTERSALT method and Kawasaki method, male had a higher estimate of 24-h urinary Na excretion (*P* < 0·001). There was no significant difference of mean 24-h urinary Na excretion between the different gender group in the Tanaka method (*P* = 0·987).

**Table 1:** Four methods to estimate 24-h urinary sodium excretion

Methods	Formula to predict 24-h urinary Na excretion(mg/d)
INTERSALT*	23 × ((25·46 + 0·46 × Na_spot_) – 2·75 × Cr_spot_ – 0·13 × K_spot_ + 4·10 × BMI + 0·26 × Age) (male)
	23 × ((5·07 + 0·34 × Na_spot_) – 2·16 × Cr_spot_ – 0·09 × K_spot_ + 2·39 × BMI + 2·35 × Age – 0·03 × Age^2^) (female)
Kawasaki*	23 × 16·3 × (Na_spot_/Cr_spot_ × PreCr)^0·5^
	PreCr = 15·12 × Weight + 7·39 × Height – 12·63 × Age – 79·9 (male)
	PreCr = 8·58 × Weight + 5·09 × Height – 4·72 × Age – 74·95 (female)
Tanaka*	23 × 21·98 × (Na_spot_/Cr_spot_ × PreCr)^0·392^
	PreCr = 14·89 × Weight + 16·14 × Height – 2·04 × Age – 2244·45
PreUNa	5·818 × Na_spot_ – 25·251 × Cr_spot_ + 46·951 × BMI – 53·817 × Age + 4912·3(male) 6·998 × Na_spot_ – 12·814 × K_spot_ + 34·009 × Height – 56·225 × Age+1522·75(female)

Na_spot_, spot urinary Na; K_spot_, spot urinary K; Cr_spot_, spot urinary creatinine; PreCr, predicted 24-h urinary creatinine; PreUNa, predicted 24-h urinary Na excretion; the units of concentration of Na_spot_, K_spot_ and Cr_spot_ are mmol/l; the unit of PreCr is mg/d. Weight and height are kg and cm. The molecule weight of Na is 23 mg/mmol.

* The equations are cited in reference 21.

**Table 2:** Differences of four methods to estimate 24-h urinary sodium excretion (g/d)

	Measured		PreUNa			INTERSALT			Kawasaki			Tanaka		
	Mean	sd	Mean	sd		Mean	sd		Mean		sd	Mean		sd
Mean														
All	3·03	1·52	3·12		0·66	2·96		1·13	43·85		16·71	21·32		6·21
Male	2·67	1·34	2·82		0·52	3·76		0·83	47·00		19·11	21·18		6·78
Female	3·41	1·61	3·43		0·64	2·15		0·74	40·64		13·15	21·46		5·58
Mean difference	Reference		0·09			–0·07			40·81			18·28		
95 % CI				–0·22, 0·04			–0·10, 0·25			39·27, 42·35			17·7, 18·83	
ICC	Reference		0·31			0·10			0·10			0·27		
95 % CI				0·22, 0·39			0·00, 0·24			0·000, 0·25			0·12, 0·39	

ICC, intraclass correlation coefficient.

The mean difference for estimating 24-h urinary Na excretion between the laboratory-based method and the INTERSALT method was –0·07 g/d (95 % CI –0·10, 0·25 g/d), while the difference between the laboratory-based method and the new method was 0·09 g/d (95 % CI –0·22, 0·04 g/d). Kawasaki and Tanaka methods highly overestimated the 24-h urinary Na excretion with the mean difference of 40·81 g/d (95 % CI 39·27, 42·35 g/d) and 18·28 g/d (95 % CI: 17·73, 18·83 g/d), respectively. The intraclass correlation coefficient for PreUNa, INTERSALT, Kawasaki and Tanaka was 0·31 (95 % CI 0·22, 0·39), 0·10 (95 % CI 0·00, 0·24), 0·10 (95 % CI 0·00, 0·25) and 0·27 (95 % CI 0·12, 0·39), respectively.

**Fig. 2:**
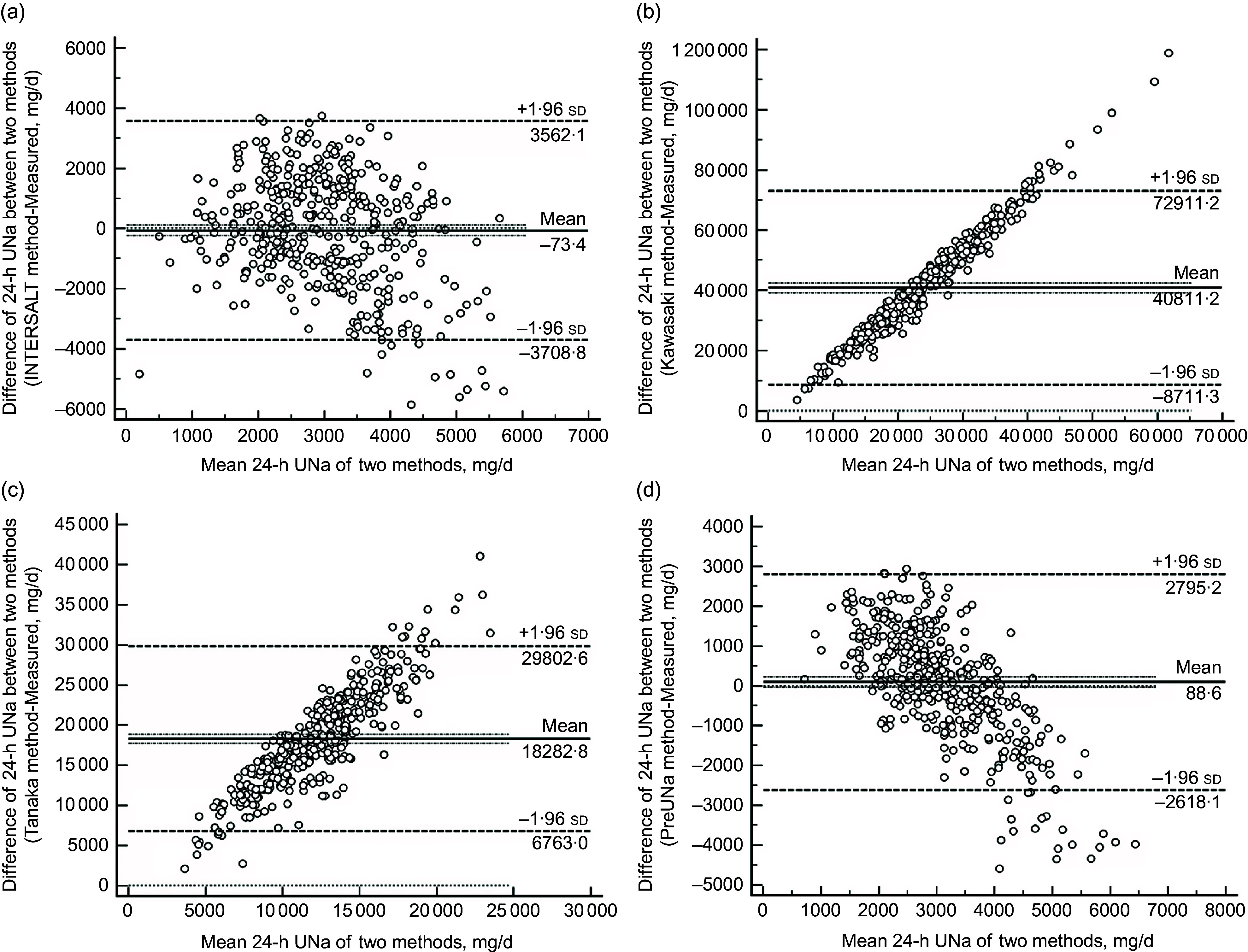
Bland–Altman plots showed the mean difference between measured and predicted 24-h urinary Na excretion of INTERSALT (a), Kawasaki (b), Tanaka (c) and PreUNa (d) methods. The long solid line represents the mean bias. The two short dotted lines represent the 95 % CI of the mean bias. The two long dotted lines represent the upper/lower limits of agreement (±1·96 sd). PreUNa: predicted 24-h urinary Na excretion

Bland–Altman plots illustrating the bias of the four methods are shown in Fig. 2. The Bland–Altman plot of the PreUNa method showed a slightly negative association between the laboratory-measured value and the predicted value. There was a strong positive association between the laboratory-measured value and the predicated value from the Kawasaki and Tanaka methods. No significant association found for the INTERSALT method.

## Discussion

This study developed a new method to estimate the 24-h urinary Na excretion using causal spot urine samples collected from an ongoing large-scale cluster randomised controlled trial. The study further evaluated the performances of the new method, INTERSALT method, Kawasaki method and the Tanaka method by comparing with laboratory-based method. The results showed that the Kawasaki method and the Tanaka method overestimated the 24-h urinary Na excretion and suggested that the INTERSALT method was the optimal choice to predict the 24-h urinary Na excretion in this study population. Despite there was a negative association found in the Bland–Altman plot of the new method, the new method had the potential to estimate 24-h Na excretion at the individual level.

The large mean differences for estimating the 24-h urinary Na excretion in the Kawasaki and Tanaka methods might be due to two primary reasons. Firstly, the Kawasaki and Tanaka methods were based on data from the Japanese population, while our research was based on northwestern Chinese population. The dietary pattern especially Na intake was different between the two populations with higher Na intake in the Chinese population. Secondly, the study participants were rural older hypertensive patients with an average less urinary volume (1 l/d), which led to a higher concentration of urinary Na.

There were many studies in the literature to evaluate the best method to estimate the 24-h urinary Na excretion using spot urine with various findings^(8–10,19–23)^. A research conducted in general Brazilian adult population found that the Tanaka method underestimated the Na intake, while Kawasaki method overestimated the Na intake. The study concluded that these two methods can estimate salt consumption when individuals had an actual salt consumption close to the population mean value (9–12 g/d for Tanaka and 12–18 g/d for Kawasaki)^(20)^. Another research conducted in the Chinese adult showed the Kawasaki method has the best performance comparing with INTERSALT method and Tanaka method^(21)^. The INTERSALT method was considered as an effective alternative method to estimate 24-h urinary Na excretion in a research of Asian adults^(17)^. However, a study in Portugal showed that neither of the methods (INTERSALT, Kawasaki, Tanaka and NHANES) were able to actually estimate the 24-h urinary Na and K excretion^(22)^. Another research involved young Black and other adults evaluated validity of predictive equations named Brown for 24-h urine Na excretion. The Brown method was gender-specific equations by using spot urine Na, K, creatinine concentrations, age and BMI. The results showed that mean biases from Brown method were not significant (–167 to 122 mg) among Blacks, while it was greatest on other adults (–247 mg, 95 % CI –348, –151 mg) when using overnight spot urine samples^(23)^. Those inconsistent findings indicated that ethnicity and the dietary pattern may be important confounding factors to predict the urinary Na excretion.

In addition, 24-h urinary Na excretion prediction was dependent on the type of the urine sample. A systematic review study compared spot, timed and overnight urine samples in estimating 24-h Na excretion from forty-three studies published from 1979 to 2010. The correlation coefficient ranged from 0·17 to 0·94, and there was no recommendation provided^(24)^. The predication of the 24-h urinary Na excretion was also dependent on the time of the spot urine samples. The INTERSALT method had the best prediction in the afternoon specimens (–90 mg; 95 % CI –208, 28 mg), while Tanaka had the best performance in the overnight specimens^(23)^. In a Chinese population study, the second morning urine samples and the post-meridiem samples were used to estimate the 24-h urine samples among 222 hypertensive patients. The study found that Kawasaki method was useful for estimating 24-h Na excretion mean levels from second morning urine samples but not reliable for estimating individual Na excretion. A recent research compared different published equations for predicting 24-h urinary K excretion and its accuracy varies by spot urine collection time, age, sex and race^(25)^. There is however currently no reliable method across all sex, race and age groups.

There were several limitations of this study. Firstly, the findings of this study were only limited to one province of the Salt Substitute and Stroke Study trial. There were five provinces involved in the Salt Substitute and Stroke Study trial. The findings to apply to the other provinces remained unknown. Secondly, there was only one spot urine sample collected in this study. Previous research found that a single spot urine sample was not a valid indicator to estimate individual level Na intake^(26)^. Thirdly, this study was limited to its sample size and study population. A large-scale study involving a heterogeneous population is needed in the future research.

## Conclusion

Compared with laboratory-measured 24-h urinary Na excretion, the predicted outcomes of the INTERSALT method and the new method showed no statistically significant bias. These two methods had the potential to estimate individual level 24-h urinary Na excretion in this study population. The extrapolation of the results needs to be careful. Future research is required to establish a more reliable method to estimate 24-h urinary Na excretion.

## Supplementary material

For supplementary material accompanying this paper visit https://doi.org/10.1017/S1368980019005019. click here to view supplementary material
