# Supplementary material for: Estimating 24-h urinary sodium excretion from casual spot urine specimen among hypertensive patients in Northwest China: the Salt Substitute and Stroke Study
Source: Public Health Nutr. 2020 Apr 29;24(4):604–10. doi: 10.1017/S1368980019005019 (PMC11574822; doi:10.1017/S1368980019005019)
Supplement: Supplementary file 1 [file S1368980019005019sup001.docx]

**Table 1 Selected characteristics of participants and urine samples test results Means ± Std / N (%)**

|  | Establish equation (n=807) | | Access equation (n=438) | | Total (n=807) | | |
| --- | --- | --- | --- | --- | --- | --- | --- |
|  | Mean/n | SD/n% | Mean/n | SD/n% | Mean/n | SD/n% | |
| Age(years) | 68.87 | 5.75 | 69.23 | 5.94 | 68.87 | | 5.75 |
| Male | 431 | 53.41 | 221 | 50.46 | 431 | | 53.41 |
| Height(cm) | 159.14 | 7.92 | 159.61 | 8.03 | 159.14 | | 7.92 |
| Weight(Kg) | 64.83 | 10.42 | 65.06 | 10.58 | 64.83 | | 10.42 |
| BMI(Kg/m^2^) | 25.55 | 3.29 | 25.49 | 3.39 | 25.55 | | 3.29 |
| SBP(mmHg) | 144.19 | 22.85 | 143.61 | 25.95 | 144.19 | | 22.85 |
| DBP(mmHg) | 84.36 | 17.18 | 84.00 | 20.01 | 84.36 | | 17.18 |
| **Casual Spot Urine** | |  |  |  |  | |  |
| Na^+^ (mmol/L) | 121.93 | 56.28 | 121.18 | 56.73 | 121.93 | | 56.28 |
| K^+^ (mmol/L) | 52.17 | 29.38 | 52.26 | 29.33 | 52.17 | | 29.38 |
| Cr (mmol/L) | 11.37 | 7.21 | 11.81 | 7.73 | 11.37 | | 7.21 |
| **24-hour Urine** | |  |  |  |  | |  |
| Na^+^ (mmol/L) | 118.25 | 50.39 | 118.26 | 51.87 | 118.25 | | 50.39 |
| K^+^ (mmol/L) | 30.63 | 15.40 | 30.24 | 14.89 | 30.63 | | 15.40 |
| Cr (mmol/L) | 7.30 | 3.75 | 7.42 | 3.72 | 7.30 | | 3.75 |
| urine volume (L) | 1.26 | 0.61 | 1.21 | 0.57 | 1.26 | | 0.61 |

SBP, systolic blood pressure; DBP, diastolic blood pressure; Cr, creatinine.

**Table 2 Variable selection results of linear regression**

| **Variables** | **Male** | | | **Female** | | |
| --- | --- | --- | --- | --- | --- | --- |
|  | **β** | **T test** | **P value** | **β** | **T test** | **P value** |
| Na_spot_ | 5.818 | 4.969 | <0.001 | 6.998 | 4.930 | <0.001 |
| K_spot_ | 0.001 | 0.001 | 0.504 | -12.814 | -4.159 | <0.001 |
| Cr_spot_ | -25.251 | -2.584 | 0.010 | -0.093 | -0.077 | 0.574 |
| Age | -53.817 | -4.443 | <0.001 | -56.225 | -4.229 | <0.001 |
| Height | 0.012 | 0.012 | 0.886 | 34.009 | 2.788 | 0.006 |
| Weight | 0.054 | 0.027 | 0.221 | 0.022 | 0.021 | 0.748 |
| BMI | 46.951 | 2.377 | 0.018 | 0.015 | 0.016 | 0.962 |
| Constant | 4912.311 | 4.648 | <0.001 | 1522.746 | 4.640 | <0.001 |

Na_spot_, spot urinary sodium; K_spot_, spot urinary potassium; Cr_spot,_ spot urinary creatinine.
